# Heterogeneous Strategies to Eliminate Intracellular Bacterial Pathogens

**DOI:** 10.3389/fmicb.2020.00563

**Published:** 2020-04-23

**Authors:** Yuan Liu, Yuqian Jia, Kangni Yang, Zhiqiang Wang

**Affiliations:** ^1^College of Veterinary Medicine, Yangzhou University, Yangzhou, China; ^2^Jiangsu Co-innovation Center for Prevention and Control of Important Animal Infectious Diseases and Zoonoses, Yangzhou, China; ^3^Institute of Comparative Medicine, Yangzhou University, Yangzhou, China

**Keywords:** antibiotic tolerance, bacterial infections, intracellular bacterial pathogens, strategies, immune

## Abstract

Antibiotic tolerance in bacterial pathogens that are genetically susceptible, but phenotypically tolerant to treatment, represents a growing crisis for public health. In particular, the intracellular bacteria-mediated antibiotic tolerance by acting as “Trojan horses” play a critical and underappreciated role in the disease burden of bacterial infections. Thus, more intense efforts are required to tackle this problem. In this review, we firstly provide a brief overview of modes of action of bacteria invasion and survival in macrophage or non-professional phagocytic cells. Furthermore, we summarize our current knowledge about promising strategies to eliminate these intracellular bacterial pathogens, including direct bactericidal agents, antibiotic delivery to infection sites by various carriers, and activation of host immune functions. Finally, we succinctly discuss the challenges faced by bringing them into clinical trials and our constructive perspectives.

## Introduction

The overuse and misuse of antibiotics have led to a global crisis of antibiotic resistance (Kupferschmidt, 2016; Yelin and Kishony, 2018; Caniça et al., 2019). For example, the emergence of “superbugs” such as methicillin-resistant *Staphylococcus aureus* (MRSA) (Lakhundi and Zhang, 2018; Turner et al., 2019), vancomycin resistant Enterococcus (VRE) (Courvalin, 2006), MCR positive Enterobacteriaceae (Liu et al., 2016), and high-level tigecycline resistance in *E. coli* (He et al., 2019; Sun et al., 2019) is accelerating and resulting in the growing failure of antibiotic treatment. Alarmingly, except for genetically encoded antibiotic resistance (Blair et al., 2015) and antibiotic heteroresistance (a transient antibiotic resistance due to gene amplifications) (Band et al., 2019; Nicoloff et al., 2019), bacteria have evolved multi-approaches to withstand antibiotic therapy such as antibiotic tolerance (Dhar and Mckinney, 2007; Kim, 2007). This is a biology phenomenon that describes bacteria that are genetically susceptible, but phenotypically tolerant to antibiotic treatment (Brauner et al., 2016). It is becoming apparent that antibiotic tolerance in bacterial pathogens plays a critical role in the relapse of many bacterial infections, particularly for chronic and recurrent infectious diseases (Grant and Hung, 2013). Notably, recent *in vitro* experiments showed that antibiotic tolerance can facilitate the emergence and evolution of resistance (Levin-Reisman, 2017). Conceivably, a better mechanistic understanding of antibiotic tolerance would give aid to developing more cost-effective coping strategies (Meylan et al., 2018).

Accordingly, several mechanisms have been demonstrated to confer antibiotic tolerance (Nguyen et al., 2011; Harms et al., 2016), including decreased metabolism, mitigation of reactive oxygen species (ROS) damage, and intracellular hiding. The activity of many bactericidal antibiotics such as β-lactam, aminoglycoside, and fluoroquinolone antibiotics mainly depends on the rapid growth or metabolism of bacteria. For example, β-lactams kill pathogens by preventing the reassembly of the peptidoglycan bonds, and eventually leading to cell death (Llarrull et al., 2010). Thus, the no-growing cells would obtain more survival advantages under exposure to β-lactams. In addition, the uptake of aminoglycosides requires the aid of proton motive force (PMF) from bacteria (Ezraty et al., 2013). Therefore, the decreased bacterial metabolisms, including tricarboxylic acid (TCA) cycle or cellular respiration, would downregulate the production of PMF and thereby confer bacterial tolerance to aminoglycosides (Allison et al., 2011; Peng et al., 2015). Furthermore, gasotransmitters such as nitric oxide (NO) and hydrogen sulfide (H_2_S) could protect bacteria against a wide range of antibiotics via mitigating oxidative stress imposed from antibiotics (Gusarov et al., 2009; Shatalin et al., 2011; Mironov et al., 2017). In addition to these tolerance mechanisms, the intracellular hiding of pathogens in mammalian cells such as phagocytes can also prevent antibiotics from killing pathogens and plays an underappreciated role in the recurrence of bacterial infections (Kamaruzzaman et al., 2017). Besides these obligate and facultative intracellular bacterial pathogens such as *Mycobacterium tuberculosis* and *Salmonella* Typhimurium (Behar et al., 2010; Xiu-Jun et al., 2010; Gengenbacher and Kaufmann, 2012), recent growing evidence demonstrated that many extracellular bacterial pathogens such as *Staphylococcus aureus* and *Escherichia coli* are able to invade, survive, and replicate in mammalian cells (Garzoni and Kelley, 2009, 2015; Foster et al., 2014). A typical example is uropathogenic *E. coli* (UPEC), which can invade bladder epithelial cells through a type 1 pilus-dependent mechanism, thus avoiding TLR4-mediated exocytic processes and eventually escaping into the cytoplasm of host cell (Anderson et al., 2004; Conover et al., 2016). It has been indicated that UPEC are by far the most common cause of urinary tract infections (UTI), which are one of the most common bacterial infectious diseases afflicting humans (Hannan et al., 2012). Importantly, these infected cells within bacteria would inadvertently act as “Trojan horses” and deliver them to non-infected tissue, then the escaped bacteria proceed to invade various other cell types and lead to recurrent infections (Tan et al., 2013). Therefore, seeking robust strategies to eliminate these intracellular bacterial pathogens are urgently needed.

In this review, we discuss our current knowledge on how these bacteria invade and survive in host cells, and how this process protects them from antibiotic killing. Furthermore, we focus our insight on these heterogeneous strategies for eliminating intracellular pathogens. Lastly, challenges and perspectives for these approaches will be highlighted.

## How Bacteria Invade and Survive in the Intracellular

**FIGURE 1:**
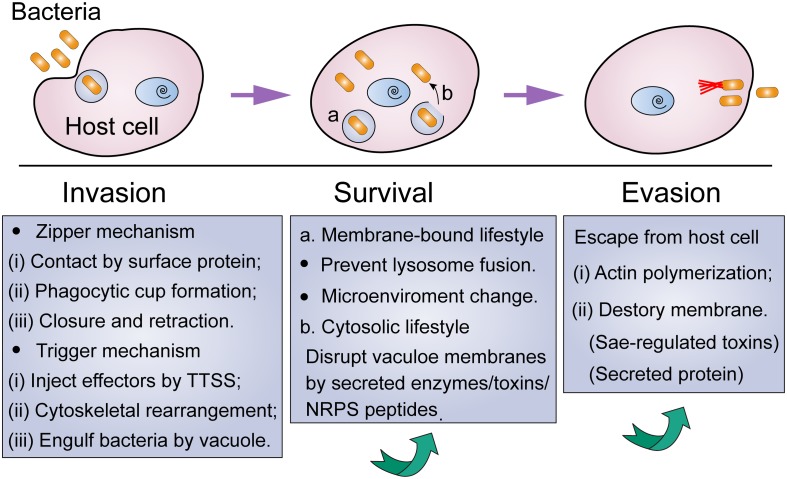
Lifestyle of intracellular bacteria from invasion to escape from host cells. Under environmental stress, non-classic intracellular pathogens tend to temporarily hide in mammalian cells via the following procedure: (i) invade these nonphagocytic cells via zipper or trigger mechanism; (ii) intracellular survival by means of membrane-bound or cytosolic lifestyle; (iii) escape from the host cell when stress disappears.

Bacterial pathogens, including extracellular bacterial pathogens, possess multiple modes of action to invade cells, and eventually evade host immune defenses and antibiotic killing (Figure 1). A better understanding of this progress would give aid to the elucidation of pathogenic mechanisms of bacteria, as well as the development of targeted prevention strategies.

### Intracellular Invasion of Bacteria

In particular, bacteria play a passive role in the phagocytosis of phagocytes. By contrast, during the invasion of non-phagocytic cells, bacteria carry out a proactive role in the interaction with the host cells (Finlay and Cossart, 1997). It has been suggested that bacteria could enter non-phagocytic cells through two major mechanisms, including zipper or trigger mechanisms (Swanson and Baer, 1995; Pascale and Sansonetti, 2004). Generally, the zipper mechanism requires the assistance of bacterial surface protein, which leads to the formation of a vacuole that could swallow the pathogens through a “zippering” process. For example, at the early stage of invasion of *S. aureus*, it adheres to endothelial cells through the interaction of fibronectin (Fn)-binding proteins (FnBPs) with α5β1 integrins on the host cell surface (Sinha and Herrmann, 2005). This interaction relies greatly on the extracellular matrix protein Fn that acts as a bridging molecule between FnBPs and integrins. Using single-cell and single-molecule experiments, Prystopiuk et al. (2018) showed that FnBPA binds to Fn via a β-zipper-like structure, and this complex in turn enhances bacterial attachment to host cells through forming a strong link with the α5β1 integrin.

The trigger mechanism is mainly mediated by the dedicated bacterial secretion systems, such as type III secretory system (T3SS) (Rosselin et al., 2011). Specifically, pathogenic bacteria such as *Salmonella* and *Shigella* could inject bacterial effectors into eukaryotic cells via the delivery of T3SS, resulting in massive rearrangement of cytoskeleton and engulfment of bacteria with entry vacuole. Different from zipper mechanism-mediated bacterial invasion, the trigger mechanism could bypass the first step of adhesion and interact directly with the cellular machinery. Interestingly, some pathogens, such as Mycobacteria, have a dual “zipper-trigger mechanism. Nevertheless, in these two invasion mechanisms, cytoskeletal rearrangement mediated by cell Rho family (Rho, Rac, and Cdc42) is necessary for bacterial invasion.

### Survival of Intracellular Bacteria

After entering mammalian cells, the next obstacle for bacteria is how to survive in the intracellular. It has been demonstrated that bacterial pathogens have developed versatile strategies to antagonize innate immune functions for intracellular survival, growth, and subsequent systemic infection (Rollin et al., 2017). Greater details on the mechanisms of intracellular survival have been emphasized and reviewed (Schulz and Horn, 2015; Cornejo et al., 2017; Niller et al., 2017). In fact, a portion of pathogens would remain in a membrane-bound compartment and adjust the subcellular location to obtain survival advantages. Instead, other bacteria would escape from the internalized vacuole and continue their normal life cycle in the cytoplasm. For example, *Salmonella* Typhimurium is a Gram-negative bacterium that can survive and replicate within host macrophages. Generally, macrophages could recognize the *S.* Typhimurium by the interaction between toll-like receptors (TLRs) and conserved microbial features such as lipopolysaccharide (LPS) and lipoproteins (Kawai and Akira, 2005). This reorganization subsequently activates antibacterial mechanisms of immune cells, including production of ROS or antimicrobial peptides (AMPs). However, *S.* Typhimurium has evolved many ways to subvert this recognition or to avoid the consequences of TLR activation (Baxt et al., 2013). For instance, the modification of lipid A by phosphoethanolamine transferase would reduce recognition by TLR4 (Reddick and Alto, 2014). In addition, an *in vivo* experiment demonstrated that TLR2 and TLR4 KO mice were highly susceptible to the intracellular bacterial pathogen *S.* Typhimurium, owing to their reduced innate immune functions (Arpaia et al., 2011). However, the deficiency of additional TLRs contributes to the elimination of intracellular bacteria through preventing the acidification of the phagosome and activation of pathogenicity island 2 (SPI-2).

Recently, it has been suggested that modulation of miRNAs also help bacterial pathogens to survive inside host cells (Das et al., 2016). MicroRNAs (miRNAs) are short non-coding RNAs that can regulate the expression of protein coding genes in eukaryotes at the post-transcriptional level. The regulation of miRNAs on eukaryotic genes includes cell proliferation, metabolic pathways, and immune response (Yao et al., 2019). For example, *M. tuberculosis* infection upregulated the expression of miR-142-3p in primary human macrophages and thus downregulated the actin binding protein *N*-Wasp, which subsequently reduced the formation of early phagosome and prevented the uptake of *M. tuberculosis* by macrophages (Bettencourt et al., 2013).

### Evasion of Intracellular Bacteria

However, for non-classical intracellular pathogens such as *S. aureus*, they would escape from the phaogosome or host cells, and lead to recurrent infections. Münzenmayer et al. (2016) investigated related factors in *S. aureus* that contribute to its escape from the phagosome or phagocytes. They found that Sae-regulated pore-forming toxins LukAB and PVL are the major factors that resulted in bacterial escape from macrophages, but were not required for the escape from HeLa epithelial cells. In addition, the Agr-regulated phenol-soluble modulins (PSMs) are mainly responsible for the evasion of bacteria from the phagosome into the cytoplasm. However, another study by Blättner et al. (2016) demonstrated that PSMs are not sufficient for the escape of *S. aureus*. They reported that the non-ribosomal peptide phevalin enhances intracellular survival of *S. aureus* and results in lung infections in a mouse pneumonia model. It is conceivable that the same bacteria may also trigger different escape mechanisms in different host cells. Despite these efforts, there is still lack of a systematic investigation on the complete life style of intracellular bacteria.

## Strategies for Eliminating Intracellular Pathogens

### Penetrating Bactericidal Agents

An inevitable barrier for anti-intracellular bacteria agents is the bacterial envelope. Screening natural products or synthetic compounds with a higher penetration ability offers an alternative approach for the eradication of intracellular pathogens (Table 1). *Salmonella* Typhimurium is an important intracellular bacteria and presents a leading cause of gastroenteritis worldwide (Tuli and Sharma, 2019). To identity potential inhibitors of intracellular *S.* Typhimurium replication, a macrophage-based chemical screening was performed. Excitingly, a psychoactive drug named metergoline exhibits potent inhibitory activity against intracellular *S.* Typhimurium by disrupting the PMF of the bacterial cytoplasmic membrane (Ellis et al., 2019). With another notorious bacterial pathogen *S. aureus*, especially the methicillin-resistant *S. aureus* (MRSA), pterostilbene, a methoxylated derivative of resveratrol originated from natural sources, was found to display bactericidal activity against it. Notably, pterostilbene could be easily engulfed by the macrophages, which in turn facilitates the eradication of intracellular MRSA (Yang et al., 2017). Another natural product is ursolic acid, derived from various plants, and which possesses many biological activities including antioxidant, anti-inflammatory, antiviral, antibacterial, and antitumoral properties. In addition to these applications, Podder et al. (2015) found that ursolic acid could activate the phagocytosis of human monocyte cells to *M. tuberculosis* through triggering the production of myeloid-relatedprotein-8 (MRP8), and significantly decrease intracellular *Mycobacterium* load through inducing the generation of ROS and NO. In addition, some cationic antimicrobial peptides (CAMPs) were reported to possess potent activity against intracellular bacteria. One example is LL-37, a principal human defense peptide, exhibited rapid and remarkable killing abilities against both extra- and intracellular *S. aureus* compared with conventional antibiotics (Noore et al., 2013).

**TABLE 1:** Representative antimicrobial agents for targeting intracellular bacteria.

Compounds	Years	Chemical structures	Pathogens	Mechanisms
Metergoline	2019	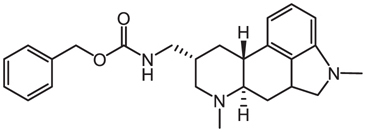	*Salmonella* Typhimurium	Disrupts bacterial proton motive force
MPepP_18_(Cu^2+^) Dimer	2018	–	*Staphylococcus aureus*	Induces intracellular ROS production
Naphtho[1,2-b]furan-4,5-dione	2017	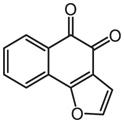	MRSA	Bacterial wall/membrane damage
Pterostilbene	2017	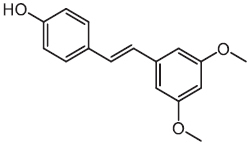	MRSA	Bacterial membrane leakage, chaperone protein downregulation, and ribosomal protein upregulation
*L*-lysine based lipidated biphenyls	2017	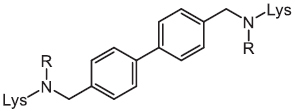	MRSA	Inhibits cell-wall biosynthesis
Polyhexamethylene biguanide (PHMB)	2016	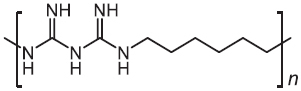	MRSA	Direct interaction with pathogens inside host cells
Thiostrepton	2015	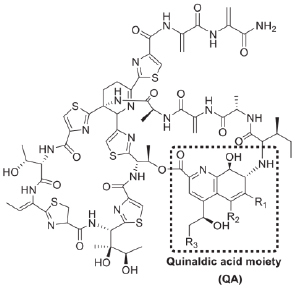	*Mycobacterium marinum*	Induces autophagy to enhance host cell defense and targets bacterial ribosome
Ursolic acid	2015	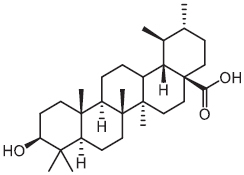	*Mycobacterium tuberculosis*	Promotes the generation of ROS and NO
LL-37	2013	–	*Staphylococcus aureus*	Membrane damage
LL-37	2010	–	*Pseudomonas aeruginosa*	Promotes the apoptosis of infected airway epithelium
AR-12	2009	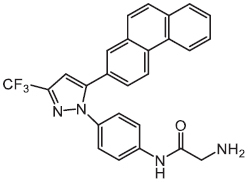	*Salmonella enterica*	Induces autophagy and inhibits the Akt kinase

*–, not shown due to the complexity of chemical structures; MRSA, methicillin-resistant Staphylococcus aureus.*

Except for natural products, a synthetic compound consists of two *L*-lysines, and lipidated biphenyls exert selective inhibition on intracellular MRSA (Ghosh et al., 2017). Mechanistic studies showed that this membrane-active antibacterial agent inhibited cell-wall synthesis, whereas the detailed mode of actions warrants more investigations. Besides, it has been shown that a synthetic cationic polymer polyhexamethylene biguanide (PHMB) killed almost 100% of intracellular MRSA strains at 4 mg/L (Kamaruzzaman et al., 2016). Of note, the uptake of PHMB is dependent on bacterial dynamin. Recently, a peptide-chlorophyll-based photodynamic therapy (PDT) agent with “sandwich” dimeric structure termed [MPepP_18_(Cu^2+^)] was fabricated (Cai et al., 2018). This new dimer induced receptor-mediated endocytosis and then efficiently eliminated intracellular *S. aureus* by the production of ROS.

Despite natural products or synthetic compounds, high permeability to cell membrane barrier is necessary for their direct antibacterial activity against intracellular pathogens. However, highly penetrating compounds are often accompanied with higher cytotoxicity in mammalian cells. Therefore, how to enable compounds to enter cells without destroying mammalian cell membranes requires a better understanding of the structure-activity relationship of drugs and innovative drug design methodology.

### Antibiotic Delivery by Various Vectors

As highly penetrating bactericidal compounds tend to be highly toxic, the delivery of low-permeability compounds into the cell by appropriate drug delivery systems may be a feasible strategy to eliminate intracellular bacteria. For instance, many hydrophilic antibiotics, such as rifampicin, exhibit weak bactericidal activity against intracellular bacteria owing to their lower permeability (Stokes et al., 2017). The conjugation of antibiotics with various vectors could contribute to delivering drugs from extracellular into intracellular. To date, antibody or nanoparticles have been tentatively utilized as effective carriers for antibiotic delivery.

#### Antibody-Antibiotic Conjugates

Antibodies such as monoclonal antibodies (mAb) have achieved profound success in disease diagnosis, small molecule detection, and cancer treatment (Brennan et al., 2010; Holford et al., 2012; Scott et al., 2012). In fact, the specific interaction of antibody-antigen has also been exploited for the treatment of various bacterial diseases. When the antigens were virulence factors or toxins secreted by bacteria, the antibody could act as an anti-virulence agent to alleviate bacterial pathogenicity. For example, a monoclonal antibody termed MAB1 demonstrated bactericidal activity against *E. coli* by inhibiting the β-barrel membrane protein folding activity, inducing periplasmic stress and disrupting outer membrane integrity (Storek et al., 2018). Nevertheless, some disadvantages, such as narrow-spectrum and concealment of essential epitopes on bacterial surfaces, may limit its success as a monotherapy to treat bacterial infections. Based on the complementary advantages of antibodies and antibiotics, we reasoned that the combination of antibody and antibiotic may display an unprecedented potential in the fight against intracellular bacterial pathogens.

**FIGURE 2:**
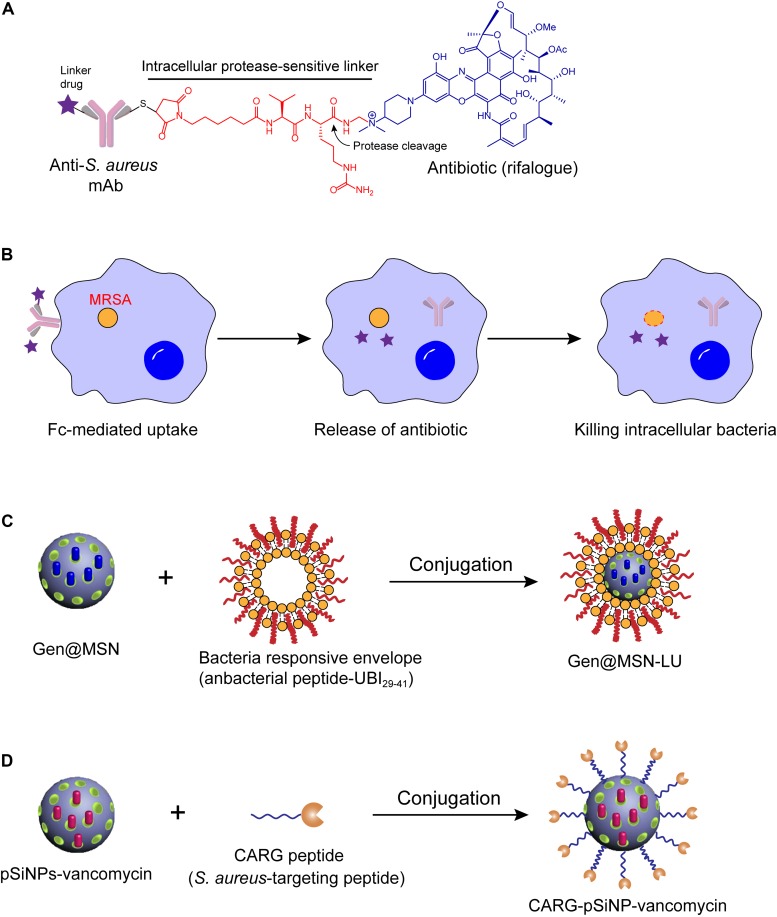
Antibiotic delivery by antibody or nanoparticle for eliminating intracellular bacteria. **(A)** Design of antibody-antibiotic conjugate (AAC). **(B)** Mechanisms of action of AAC. **(C,D)** Schematics of the synthetic route of Gen@MSN-LU **(C)** and CARG-pSiNP-vancomycin **(D)**.

A recent meaningful example was the antibody-antibiotic conjugate (AAC) approach. To eliminate the intracellular *S. aureus*, an antibody–antibiotic conjugate (AAC) consisting of an anti-*S. aureus* antibody and a highly effective antibiotic rifalogue (a rifampicin derivative) via a cathepsin-cleavable covalent linker was developed (Figure 2A; Lehar et al., 2015). This idea may have been inspired by the antibody–drug conjugate (ADC) concept that has been successfully applied in the treatment of cancer (Chari et al., 2014). Noteworthy, this AAC conjugate is inactive before entering the host cells, which is likely to reduce both the emergence of antibiotic resistance (by reducing the exposure of other bacteria to the active drug) and the disruption of the body’s normal communities of microorganisms. However, after the conjugate enters into the host cells by specific binding of antibody and antigen, the endoenzyme will destroy the chemical bridges and the active antibiotic form will be subsequently released (Figure 2B). Strikingly, in a mouse infection model, this AAC was much more effective in reducing pathogen loads than two conventional antibiotics that are currently used to treat intractable *S. aureus* infections (Mariathasan and Tan, 2017). Importantly, the AAC (DSTA4637S developed by Roche) has reached phase I trials (NCT02596399) (Beck et al., 2017; Carter and Lazar, 2017).

Although AAC strategy displayed huge potential in eliminating intracellular bacteria, there are still some probable limitations that prevent it entering into clinic. For instance, whether AACs are efficient at treating bacterial infections in humans as well as in mice, or whether antibodies produced by the patients themselves will occupy the antigenic determinant of bacteria and interfere with the targeting of the AAC. Also an issue could be whether the whole AAC would be recognized as an antigen in human beings and produce immunogenicity. We hope that these challenges could be successfully addressed, which would be helpful in accelerating this promising therapy. In addition, it is exciting to develop more AACs that target other clinically relevant pathogens, particularly for Gram-negative bacteria.

#### Nanoparticle-Based Carriers

The combination of nanoparticle delivery with specific antibiotics also provides a particularly powerful means for improving drug efficacy. During the past decades, nanoparticles such as liposomes, biodegradable polymeric nanoparticles, inorganic nanoparticles, and mesoporous silica nanoparticles have been widely studied as the drug-carriers of antimicrobial agents for the treatment of intracellular bacteria (Goyal et al., 2016). For example, the rifampicin-loaded liposomes inhibited the growth of Mycobacterium avium complex (MAC) in the infected macrophages (Zaru et al., 2009). Besides, a highly hydrophobic citral-derived isoniazid analog (JVA) was enfolded with poly-lactide-coglycolic acid (PLGA) nanoparticles, thus inhibiting *M. tuberculosis* growth by enhancing intracellular drug bioavailability (Faria et al., 2012). *Klebsiella pneumoniae* is considered as a foremost Gram-negative pathogen in life-threatening nosocomial pulmonary infections (Weinstein et al., 2005). Alarmingly, this pathogen remains viable within vacuolar compartments after being phagocytosed by macrophages (Cano et al., 2015), which results in chronic infection. To address this problem, gentamicin-loaded PLGA nanoparticles (GNPs) were performed via a water-in-oil-in-water formulation process. Evidence showed that GNPs would be phagocytosed by *K. pneumoniae* infected macrophages, and remarkably reduce the intracellular bacteria without stimulation of pro-inflammatory pathways (Jiang et al., 2018). Another nanocarrier that utilized mesoporous silica nanoparticle (MSN) as a template was arginine grafted mesoporous silica nanoparticle (Arg-MSN) (Mudakavi et al., 2017). For example, ciprofloxacin-loaded Arg-MSN (Cip Arg-MSN) displayed two-fold higher antibacterial activity against intracellular *Salmonella* than ciprofloxacin alone treatment. In addition, a gold nanoparticle-DNA aptamer (AuNPApt) conjugate-based delivery system was employed for the delivery of AMPs into mammalian living systems. Consequently, *C*-terminally hexahistidine-tagged A3-APO was efficiently delivered and exhibited the highly potent ability to eliminate intracellular *S*. Typhimurium by disrupting the bacterial membrane (Yeom et al., 2016).

However, their lack of specific targeting to the infected tissue or pathogens reduces the therapeutic efficacy of the encapsulated antibiotics and promotes the evolution of drug resistance. To improve the target specificity, a unique intracellular antibiotic delivery nanoparticle was composed of multi-chemical groups, including a core gentamicin-mesoporous silica nanoparticle, an infected microenvironment-sensitive lipid bilayer, and bacteria targeting moieties ubiquicidin (UBI_29–41_) (Figure 2C; Yang et al., 2018). These distinctive compositions endowed the complex with higher specificity and sensitivity of antibacterial drugs to the infection sites and allowed for sufficient accumulation. Consistently, rapid drug release and reduced *S. aureus* loads were observed both *in vitro* and *in vivo* intracellular infection models. In another study, a cyclic 9-amino-acid peptide CARGGLKSC (CARG) was identified through phage displaying, which showed specificity binding activity to *S. aureus*, but not for other bacteria (Figure 2D). Because of the infection targeting effect of CARG, the conjugation of vancomycin-loaded nanoparticles and CARG selectively accumulates in *S. aureus*-infected tissues in mice models and improves survival of mice (Hussain et al., 2018). With the need for precise treatment and control of bacterial resistance, nanoparticle-based carriers with higher specificity would be more prevalent. Rational design of bacteria identification moieties in the process is particularly important.

### Activation of Host Immune Functions

Host cells provide a physical barrier for the protection of intracellular bacteria against antibiotic killing. It is conceivable that the activation of host immune functions would offer a promising approach to eliminate these intracellular pathogens. Infiltrated phagocytes kill the invading pathogens via either oxygen-dependent or -independent bactericidal system, represented by ROS and reactive nitrogen species (RNS), respectively, and via bactericidal secreted protein and AMPs such as β-defensins. However, some obligate intracellular bacteria, such as *M. tuberculosis*, can still live within macrophages owing to their ability to arrest phagolysosome biogenesis. In addition, the above bactericidal mechanisms are not applicable for other non-professional immune cells. Alternatively, host cells can protect against intracellular pathogens by activating the autophagy innate defense system or by initiating apoptosis.

#### Autophagy

Recently, growing evidence has demonstrated that autophagy provides a universal protective strategy for host cells against a variety of intracellular pathogens, including obligate intracellular bacteria (*Listeria monocytogenes*, *Shigella flexneri*, *Salmonella* Typhimurium, and *M. tuberculosis*) and extracellular pathogens (Huang and Brumell, 2009, 2014; Randow and Youle, 2014).

**FIGURE 3:**
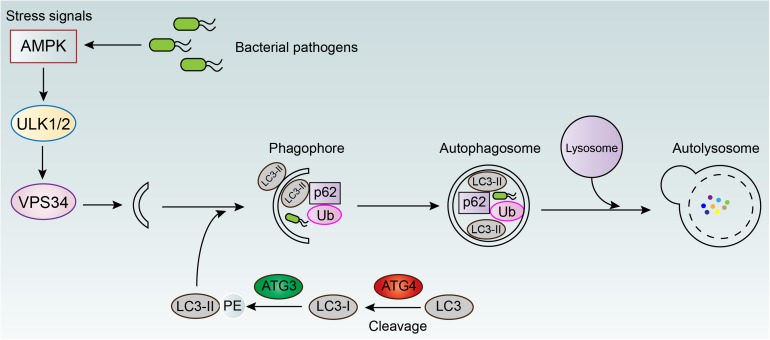
Autophagy mediated elimination of intracellular bacterial pathogens. AMPK, adenosine 5’-monophosphate (AMP)-activated protein kinase; ULK1/2, unc-51 like autophagy activating kinase 1/2; VPS34, vacuolar protein sorting 34; LC3, light chain 3; ATG3/4, autophagy-related protein 3/4; p62, ubiquitin-binding protein 62; Ub, ubiquitin.

Autophagy is an evolutionarily conserved catabolic mechanism that maintains cytoplasmic homeostasis by inducing the degradation of damaged organelles or misfolded proteins (Levine and Kroemer, 2008; Mizushima et al., 2008). It has been demonstrated that autophagy can be activated by multiple factors, including pharmacological agonists (e.g., rapamycin) and physiological signals (e.g., starvation), as well as Toll-like receptor ligands and inflammatory cytokines (e.g., IFN-γ and TNF) (Delgado et al., 2009). Meanwhile, the activation of autophagy can be employed by host cells for combating various intracellular bacteria. Initiation of autophagy is usually characterized by the formation of microtubule-associated protein light chain 3 (LC3) puncta, as well as the conversion of LC3-I into its lipidated form (LC3-II) (Yoshimori, 2004). Subsequently, autophagosomes (a double-membrane compartments) around targeted bacteria are formatted and transport the bacteria to lysosomes for degradation (Figure 3). For instance, Singh et al. (2006) demonstrated that murine Irgm1 (LRG-47) guanosine triphosphatase activated autophagy and produced large autolysosomal organelles, which contribute to the elimination of intracellular *M. tuberculosis*. The mammalian intestine is colonized with a diverse community of bacteria including commensals bacteria, which perform many beneficial functions in host metabolism and digestion. Therefore, defense against these invasive bacteria is critical for intestinal homeostasis. Benjamin et al. (2013) demonstrated that intestinal epithelial autophagy is essential for this process, which required epithelial cell intrinsic signaling via the innate immune adaptor protein MyD88. In addition, the effect of autophagy on preventing intracellular bacterial infections was further investigated in two infection models, including *Caenorhabditis elegans* and *Dictyostelium discoideum* (Jia et al., 2009). In both organisms, genetic inactivation of the autophagy related pathway promoted bacterial intracellular replication and decreased animal lifespan, suggesting the essential role of autophagy in host defense *in vivo*.

Interestingly, some previous studies have suggested that miRNAs carry out an important role in modulating cell autophagy (Taganov et al., 2007). For example, microRNA-155 was found to promote the process of autophagy by targeting the negative regulator of autophagy, Ras homolog enriched in brain (Rheb), and thus decreased the intracellular mycobacteria (Jinli et al., 2013). However, some bacteria have developed unique strategies to escape from autophagy mediated elimination (Baxt et al., 2013), including inhibiting autophagy related signaling pathways (Shahnazari et al., 2011; Tattoli et al., 2012), avoiding host cell recognition (Ogawa et al., 2005; Yoshikawa et al., 2009), interfering with the autophagy pathway (Augustine et al., 2012; Dong et al., 2012), or blocking fusion of the autophagosome with the lysosome (Chargui et al., 2012). However, the subversion mechanisms of autophagy by bacteria are still not fully understood. Nevertheless, these findings inspire a new approach to activate autophagy and eliminate intracellular bacteria by modulating the expression of some specific miRNAs.

Collectively, excluding some special cases, these results implied that the activation of autophagy by the addition of exogenous compounds may give aid to overcoming intracellular bacteria. For instance, a novel small-molecule agent AR-12 could promote autophagy in macrophages, evidenced through the increased autophagosome formation, and thereby inhibit the intracellular bacterial growth of *Salmonella* Typhimurium (Chiu et al., 2009). Another existing example is thiostrepton (TSR), which is an archetypal thiopeptide antibiotic possessing a quinaldic acid (QA) moiety in the side ring system (Bagley et al., 2005; Zhang and Liu, 2013). A previous study demonstrated that thiopeptides kills bacteria by targeting bacterial ribosome (Morris et al., 2009). Zheng et al. (2015) showed that thiostrepton (an archetypal thiopeptide antibiotic) and its derivatives enhanced host cell defense through inducing ER stress-mediated autophagy. Consequently, thiopeptide antibiotics are effectively killing the intracellular pathogen *Mycobacterium marinum*. This finding also suggested the dual action of antibiotics, including direct antibacterial activity and immunoregulatory function, would be more potent in the fight against bacterial infections.

#### Apoptosis

**FIGURE 4:**
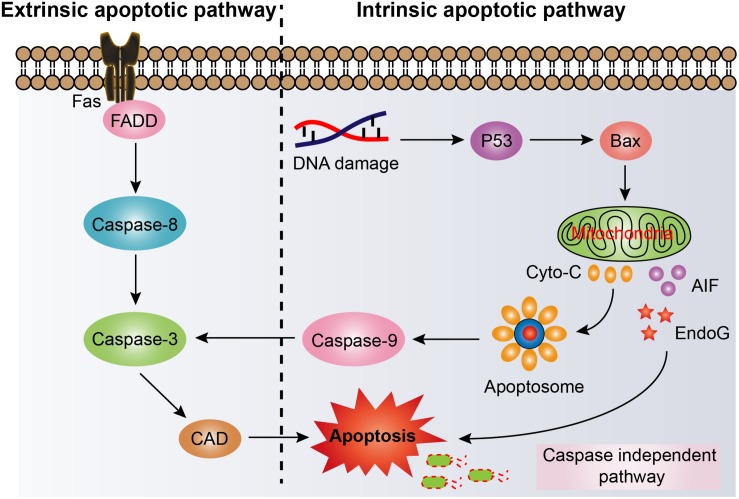
Activation of cell apoptosis pathway in host cells for eliminating intracellular bacterial pathogens. Fas, tumor necrosis factor superfamily receptor member 6; FADD, Fas-associated death domain; Caspase, cysteinyl aspartate specific proteinase; CAD, caspase activated DNase; P53, protein 53; Bax, bcl-2 Assaciated X protein; Cyto-C, cytochrome C; AIF, apoptosis inducing factor; EndoG, endonuclease G.

Another protective strategy for host defense is cell apoptosis. Apoptosis, also named type I PCD, is an ordered and evolutionary conserved cellular process that occurs in various pathological and physiological conditions (Hengartner, 2000). Apoptosis is generally characterized by specific morphological and biochemical changes of dying cells. Morphological changes include cell shrinkage, nuclear pyknosis and fragmentation, the formation of dynamic vesicles, and the loss of adhesion to adjacent cells or extracellular matrix (Allen et al., 1997). Biochemical changes include the cleavage of chromosome DNA into nucleosome fragments, the externalization of phosphatidyl serine, and the cleavage of some intracellular substrates (Allen et al., 1997; Elmore, 2007). Apoptosis could be majorly divided into two pathways based on the activation manner of caspases, including the extrinsic (also called death receptor) pathway and the intrinsic (also called mitochondrial) pathway (Ichim and Tait, 2016). The extrinsic apoptotic pathway is activated by extrinsic stimulations via plasma membrane death receptors such as tumor necrosis factor-related apoptosis-inducing ligand receptors (TRAILR) or FAS or TNF receptor (Li et al., 1998; Micheau and Tschopp, 2003; Kimberley and Screaton, 2004). After ligand binding, death receptors activate caspases, leading to widespread cleavage of caspase substrates and rapid cell death (Taylor et al., 2008). By contrast, the intrinsic pathway is engaged by intercellular stimuli, including DNA damage, endoplasmic reticulum (ER) stress, and production of ROS (Czabotar et al., 2014). Importantly, these stresses would then increase permeabilization of mitochondrial outer membrane and dissipate membrane potential (Tait and Green, 2010). The released endonuclease G (EndoG) or apoptosis inducing factor (AIF) would directly bring about apoptotic death by caspase-independent pathway (Li et al., 2001; Xiaochen et al., 2002). Whereas the cytochrome C from disrupted mitochondria would trigger the formation of apoptosome (Riedl and Salvesen, 2007), which activates the caspase-9 and eventually induce caspase-dependent intrinsic apoptotic pathway (Figure 4).

As the understanding of the critical role of apoptosis in host defense increased, the potential of activation of apoptosis in preventing intracellular bacteria was gradually understood. For instance, overexpression of EBP50 (a Na^+^/H^+^ exchange regulatory factor) significantly promoted the elimination of intracellular *M. tuberculosis* through increasing level of apoptosis in macrophages. In contrast, virulent *M. tuberculosis* have the capacity to escape the macrophages killing by interfering with the function of EBP50 (Guo et al., 2016). Another example showed that pharmacological inhibition or genetic deletion of the host cell pro-survival protein BCL-XL induced intrinsic apoptosis of infected macrophages with virulent *Legionella* strains, thereby abrogating *Legionella* replication (Speir et al., 2016). Notably, the cationic host defense peptide LL-37 was also found to promote apoptosis of infected airway epithelium except for its direct bactericidal activity, which increased the pulmonary clearance of the opportunistic pathogen *Pseudomonas aeruginosa in vivo* (Barlow et al., 2010).

## Challenges and Perspectives

**FIGURE 5:**
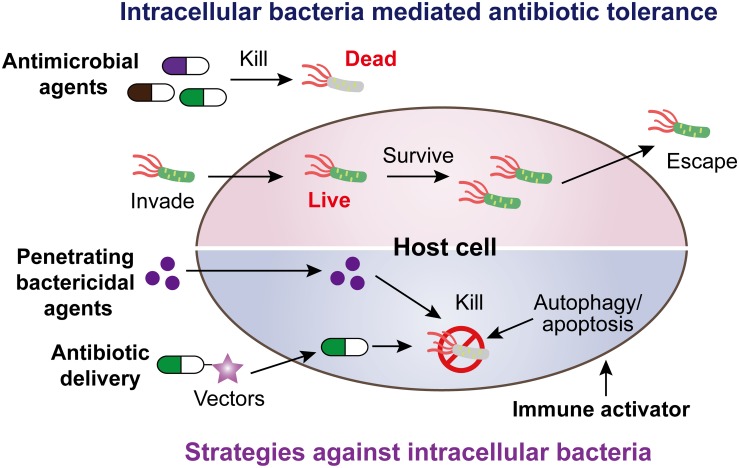
Scheme of intracellular bacteria mediated antibiotic tolerance and potential coping strategies.

Intracellular bacterial pathogens mediating antibiotic tolerance pose a growing threat for global heath. Alarmingly, these pathogens include obligate intracellular and extracellular bacteria that have evolved various modes of action to invade, survive, and escape from host cells. There is an unmet and urgent need to seek feasible strategies to counter this crisis. In this review, we summarize our current knowledge about heterogeneous strategies with a high potential to eliminate intracellular bacterial pathogens, including penetrating bactericidal agents, antibiotic delivery by various vectors, and activation of host immune functions (Figure 5).

Although these strategies demonstrated potential efficacy, there are still many challenges to hinder their successful application in the clinical setting. For example, the potential toxicity of penetrating bactericidal agents to normal host cells due to their strong membrane damage. As for antibiotic-adjuvants conjugates, the stability and potential antigenicity in the human body remain a concern. Similarly, the limitation for the immunomodulation approach is the need to balance between activation of host immune and increased elimination of intracellular pathogens, which otherwise may result in more allergic reactions owing to the overactivation of host immune. Recently, some machine-learning methods or computer-assisted designs have been devised for optimizing the structure-activity of drugs and decreasing the side effects (Zhang et al., 2017a; Vamathevan et al., 2019). In addition, other strategies such as bacteriophage can also provide a complement approach (Kutateladze and Adamia, 2010; Cisek et al., 2017). For instance, virulent bacteriophage vB_SauM_JS25 could penetrate bovine mammary epithelial cells and clear intracellular *Staphylococcus aureus* in a time-dependent manner (Zhang et al., 2017b). Taken together, despite these obstacles, various strategies presented in this review offer promising pipelines to address the clinical infectious diseases caused by intracellular bacteria.

## Author Contributions

All authors listed have made a substantial, direct, and intellectual contribution to the work, and approved it for publication.

## Conflict of Interest

The authors declare that the research was conducted in the absence of any commercial or financial relationships that could be construed as a potential conflict of interest.
